# Nature benefits revisited: Differences in gait kinematics between nature and urban images disappear when image types are controlled for likeability

**DOI:** 10.1371/journal.pone.0256635

**Published:** 2021-08-27

**Authors:** Daria Burtan, Jeremy F. Burn, Ute Leonards

**Affiliations:** 1 School of Psychological Science, University of Bristol, Bristol, United Kingdom; 2 Queen’s School of Engineering, University of Bristol, Bristol, United Kingdom; University of New England, AUSTRALIA

## Abstract

Exposure to urban environments requires more cognitive processing than exposure to nature; an effect that can even be measured analysing gait kinematics whilst people walk towards photographic images. Here, we investigated whether differences in cognitive load between nature and urban scenes are still present when scenes are matched for their liking scores. Participants were exposed to images of nature and urban scenes that had been matched *a priori* for their liking scores by an independent participant sample (n = 300). Participants (N = 44) were either asked to memorise each image during walking or to rate each image for its visual discomfort after each walk. Irrespective of experimental task, liking score but not environment type predicted gait velocity. Moreover, subjective visual discomfort was predictive of gait velocity. The positive impact of nature described in the literature thus might, at least in part, be due to people’s aesthetic preferences for nature images.

## Introduction

Exposure to nature is generally agreed to have a positive impact on many aspects of physical and mental health [1–3]. Overwhelming amounts of evidence support the claim that interacting with nature requires lower cognitive input than interacting with urban environments [4–10]. Even simple exposure to images of nature environments as compared to images of urban environments improves performance on attentional tasks [11, 12] or modulates gait kinematics [13], further supporting the notion that at least some of these beneficial effects of nature are due to processing differences in visual cognition required for the two environments. Yet, it remains unclear whether cognitive load differences between nature and urban environment types are due to low-level visual features (such as image statistics), high-level visual features (e.g. objects, larger shapes), attention, semantic associations with image types, differences at the category level (i.e. the environment itself), or any kind of interaction between these factors.

Also, aesthetic preference for nature environments has been considered to impact cognitive processing benefits that exposure to such environments may give us (e.g. [14]). For example, exercising whilst viewing photographs of pleasant rural environments was more effective in reducing blood pressure than exercising whilst viewing photographs of pleasant urban environments, unpleasant rural and unpleasant urban environments [1]. In the study presented here, we therefore controlled for any potential aesthetics confound by selecting pairs of images in which nature and urban image had similar aesthetic rating scores.

Neuropsychological and neuroimaging studies demonstrate that aesthetic experience arises from interactions between three neural systems: sensory-motor, emotion-valuation and meaning-knowledge systems [15]. According to the Aesthetic Triad Model (ATM) by Chatterjee and Vartanian [15], aesthetics is a term that describes the experience of interactions with objects/scenes that evoke emotions associated with the reward system of “liking” or “pleasure”. Aesthetic preference was defined as aesthetic liking established on the basis of ratings for liking. Ratings of liking are a good measure to capture participants’ aesthetic response according to work by Graf and Landwehr based on the Pleasure-Interest Model of Aesthetic Liking (PIA Model; [16]). The model distinguishes between two positive aesthetic responses: pleasure and interest; raising the question of what people really mean when they talk about nature being more aesthetically pleasing than urban environments and how much this depends on their definitions of both aesthetics and nature, respectively. More recent research provides empirical evidence that both pleasure and interest are triggered by similar processing dynamics, strongly suggesting that both aspects of “aesthetic liking”, aesthetic pleasure and aesthetic interest, are based on a single mechanism [17]. We therefore decided that for our image selection it should not matter what drove people’s liking responses–interest or pleasure.

A large body of work investigating the impact of nature exposure on attention and memory performance is based on two theoretical perspectives in environmental psychology. Firstly, Attention Restoration Theory (ART; [5, 6, 18, 19]) proposes that exposure to nature allows us to replenish our attentional resources whilst exposure to urban environments exhausts directed attention mechanism, and this, in turn, fatigues the brain. In particular, Kaplan and Kaplan proposed four predictors for an environment’s ability to be restorative: soft fascination (i.e. the interest generated by a scene and its informational richness), extent (the connection between individual elements within an environment), being away (the ability to allow one’s mind to wander away from every-day issues) and compatibility (the extent to which environmental features meet one’s preferences) [19]. Secondly, Stress Recovery Theory (SRT; [9, 10]) suggests that spending time in nature evokes positive affective responses, and thus fosters faster recovery from physiological stress. Reduced stress levels, then, lead to improved performance on cognitive/attentional tasks. Whilst ART thus focuses predominately on a cognitive explanation of the nature benefit, SRT highlights the importance of affective processes impacting cognition. Such positive affective responses to nature environments but not urban environments have been repeatedly linked to increased aesthetic preferences for nature. Images, slides, videos have been used as stimulus material in these studies [18, 20–27]. The link between aesthetic preference and ART is less conclusive. A meta-analysis of 61 studies by Stamps [28] revealed that Kaplan and Kaplan’s [18] four informational variables coherence (immediate understanding), complexity (immediate exploration), legibility (inferred understanding) and mystery (inferred exploration) neither predicted aesthetic preference reliably nor did these four variables jointly distinguish between nature and urban environments. Note, however, that some of the individual environmental factors, such as the restorative factor fascination and the informational factor coherence, were found to account for some of the variance in participants’ scene preference ratings (e.g. [29, 30]), in particular for built environments (e.g. [31]).

Evolutionary theories suggest that humans are innately attracted to nature as they evolved in natural settings (Biophilia Hypothesis [32, 33], SRT [9]). Other more physiologically oriented theories such as the so-called Perceptual Fluency Account (PFA) suggest that aesthetic preference for nature is a function of processing fluency [34–37]. This theory is based on assumptions that objects differ in their low-level sensory features, and some of these features are easier to process than others (e.g. symmetry). PFA has been supported by research in computer-generated imagery which found, for example, that exposure to high fractal content results in more fluent visual processing [37]. Fractals are self-repetitive patterns across different spatial scales [38], and they are used as a quantitative measure of visual complexity (e.g. [39]). Nature images have greater amounts of fractals than urban images [40], and fractal properties have been found to be crucial visual drivers of positive response to nature [41], giving rise to the hypothesis that this particular image characteristic may be part of the basis for preference.

Image statistics not only indicate liking but also how uncomfortable an image is to look at, with urban scenes being usually perceived as more uncomfortable to look at than images of nature scenes [40]. Visual discomfort is a measure of visual stress related to uncomfortable and unpleasant physiological symptoms reported during exposure to certain visual stimuli [42]. Symptoms might include headache, visual distortions, illusions, nausea or blurred vision. Visual discomfort seems to be associated with image statistics, with images diverging further from a 1/frequency (1/f) contrast distribution inducing increased cortical responses, assumed to underlie increased visual discomfort [43, 44]. Visual discomfort of built environments has been found to increase the more their image properties deviate from image properties typical of nature images [45]. Images of urban environments are diverging further from a 1/f contrast distribution than images of rural environments [46], which might partly explain why urban images are more uncomfortable to look at than nature images (e.g. [40]). Moreover, visual discomfort and its interaction with the environment (urban vs. nature) predict gait changes, with people walking more slowly towards images of urban scenes as the ones more uncomfortable to look at [13]. Though related, the exact link between aesthetic preference and visual discomfort remains unclear. Judgements of aesthetic preferences express appreciation of an object, and are associated with pleasure [15, 47]. Visual discomfort, in contrast, is defined as uncomfortable and unpleasant feelings related to physiological symptoms.

What do we mean when we talk about nature environments as opposed to urban environments? Bratman, Hamilton, & Daily [14] proposed a system of categorization for different types of landscapes based on the analysis of stimulus sets used in studies that investigated the psychological or behavioural impact of nature images, which facilitates the definition of ambiguous landscapes incorporating both flora and man-made objects. In their model, nature was defined as an area that contains elements of living systems, including plants and non-human animals, of different degrees of human management, implying that both “pristine wildness” and “urban parks” are a part of nature. Our nature images followed this definition and were entirely free of human artefacts such as buildings as well as of animals. Note, however, that some of our urban images, whilst all dominated by buildings, included partially visible blue-green infrastructure. Please also note that aesthetic judgements of landscapes vary across cultures (e.g. [48–50]) and personality factors (e.g. [51]).

The aim of the current study was to investigate whether differences in cognitive processing load between nature and urban scenes remain when each urban scene presented matches the liking score of a nature scene presented in the same image set; in other words, when the range and values of liking scores are identical between the two image types, controlling for likeability across the entire image set. To quantify cognitive load differences, we measured differences in human gait kinematics, as gait is sensitive to changes in cognitive load: increased cognitive load leads to decreased walking speed and increased gait variability [52]. Changes in gait kinematics seem sensitive enough even to reflect the amount of cognitive load required to process any perceived nature or urban image whilst walking, on a trial-by-trial basis: in an earlier study, we found that exposure to urban scenes decreased gait speed and step length compared to exposure to nature scenes [13]. Whilst the images used in this earlier study were closely controlled for image configuration between different environments, they had not been controlled for liking. Here, we hypothesised that differences in gait kinematics between walks toward nature and urban images would not arise if these two image categories were matched a priori in pairs for their liking scores; in other words, the nature benefit should be abolished if nature and urban environments are similarly aesthetically pleasing. As in our earlier study [13] subjective visual discomfort induced by environment type predicted gait changes, we further hypothesised that differences in subjective visual discomfort between nature and urban scenes, even if controlled for aesthetics, would still differentially affect gait kinematics. The second goal of the current study was therefore to investigate whether subjective visual discomfort and image statistics (fractal dimension) contribute to the cognitive load differences between environment types when aesthetics is controlled for. We investigated these ideas with a dual-task paradigm. Participants walked toward urban or nature images controlled for aesthetic preference whilst performing a cognitive task to ensure that they engaged with the images shown. This cognitive task could either be a memory task or a visual discomfort rating task. Images for this study were a subset of images from a large image set for which liking scores had been obtained for each individual image by an independent participant sample. This allowed us to select image pairs of urban and nature scenes with similar liking scores. If reports in the literature of a positive impact of nature exposure on attention and memory were due to increased aesthetic preferences inherent in the nature environments used, then, theoretically, such differences should not arise or be substantially smaller if nature and urban image categories are matched in pairs for their liking scores.

## Materials and methods

### Participants

Sample size calculations took into account the substantial amount of repetitions within individual participants for the conditions of interest (environment type), and were based on modelling estimates for within-participant repeated measures correlations provided by Bakdash & Marusich [53]: to obtain 80% power for a medium effect size (0.3) and within participant repeated paired measures of 20 or more, we needed a minimum of 12 participants. As the cognitive task differed between participants, we doubled the number of participants per cognitive task to account for possible task-specific effects.

Fifty participants (43 females and 7 males, aged between 18 and 61 years, mean age 21 years +- 6.6 SD) took part in this study. Twenty-seven (24 females and 3 males, aged between 18–61 years, mean age = 22, SD = 8.26) of them were asked to walk toward images presented at the back wall of the lab whilst having to memorise the presented images (i.e. walking whilst performing a memory task), whilst the other twenty-three participants (19 females and 4 males, aged between 18–35 years, mean age = 21, SD = 3.86) were asked to walk toward the same images and to rate each image for visual discomfort (i.e. walking whilst performing a visual discomfort rating task). Participants were randomly assigned to the two tasks.

All participants reported normal or corrected-to-normal visual acuity as well as no neurological conditions that could affect their walking. They also confirmed that they were healthy and fit enough to walk without difficulties for an hour. All participants were provided with written and verbal information about the study and signed the consent form prior to their experimental session. They were also given information about the right to withdraw and possible breaks to take during the experimental session whenever they felt needed. Participants were either volunteers or took part in the experiment for course credit. The experiment was approved by the Faculty of Life Sciences’ Ethics Committee at the University of Bristol (ref. 20111878362).

### Stimuli

The stimulus set contained 50 images of nature and 50 images of urban scenes matched beforehand by an independent participant sample for their liking scores: images had been selected from a larger image set of 400 images in which every single image had been rated for likeability on a 7 point Likert scale by an independent observer sample (See S1 File). This larger image set included images of the “places” category of the scene recognition database [54], in addition to photographs of landscape and urban spaces taken in Europe and Australia by the authors. Images presented environmental scenes without people. The subset of stimuli matched for liking scores used here contained 50 nature-urban image pairs with average liking scores ranging between 2.82 and 5.61 on the above-mentioned 7-point Likert scale. Results of Independent Samples t-test confirmed that there was no significant difference in average liking scores between these nature and urban images (Nature: M = 4.22, SD = .67, Urban: M = 4.22, SD = .67, t(98) = .001 p > .05, mean difference = 0.00013).

Fractal dimensions were calculated for each image, based on the Minkowski–Bouligand fractal dimension box-counting technique [55]. For this, images had been normalised and converted to greyscale. Prior to running the actual box counting algorithm, images were binarized using the mean image value that calculates fractal dimensions based on a variety of different box sizes. An Independent Samples t-test revealed that, as expected, fractal dimensions in nature scenes (M = 1.65, SD = 0.13) were significantly higher than in urban scenes (M = 1.59, SD = 0.14), t(98) = 3.567, p < 0.01).

### Tasks and procedure

At the beginning of the study, participants were provided with written and verbal information about the study and descriptions of the respective motor-cognitive task they were asked to perform. Participants were asked to wear an elasticated belt at hip height with three small spherical retro-reflective markers to locate the left hip, right hip and lower abdomen (hereon referred to as hip). We also attached markers to participants’ shoulders (lateral clavicle), knees (patella), outside of their ankles (lateral malleolus), and their feet (first metatarsal-phalangeal joint). The location of these markers was detected during walking by a motion capture system (Oqus, Qualisys AB, Sweden) consisting of 12 cameras (x-direction depicting lateral movement, y-direction depicting direction of travel down the laboratory, z-direction depicting vertical movement).

The system was calibrated prior to each individual experimental session, providing an accuracy of 1mm^3^ for reflective markers and a recording frequency of 100Hz. The capture space was 12m x 2m x 2.4m. An Optoma EW536 projector displayed the visual stimuli during the experiment session in dim lighting condition onto the far wall of the laboratory.

Participants were asked to walk repeatedly down the laboratory, as soon as an image appeared on the wall at its far side (15m), from the marked starting cross location to the marked ending cross location; each walk counting as one trial. Participants were asked to walk in their natural walking speed in the straightest possible way.

Each participant engaged in 105 trials. For each trial, one of the following 105 photographs was displayed in random order: a nature scene (50), an urban scene (50), or a blank grey image as a control condition (5). Participants in the “walking whilst performing a memory task” condition were required to memorise each image during walking and return to the starting position for the next walking trial (note that task compliance for this condition was checked in a separate behavioural experiment after the actual walking experiment had been finished–see below for further information). Participants in the “walking whilst performing a visual discomfort rating task” were required to verbally rate each image for its visual discomfort on a 7-point Likert Scale (from 1 = extremely comfortable to view to 7 = extremely uncomfortable to view; 4 = neither comfortable nor uncomfortable) after each walk and then return to the starting position. Their response was noted by the experimenter.

The image display size was 3m wide x 2m high, corresponding to 11.4° x 7.6° of visual angle when viewed from the walking start point, and 57° x 38° of visual angle when viewed from the end line of the 3D motion capture space. Once participants had stepped on the ending cross location, they were asked to come back to the start point. Participants were offered two breaks during the session (after trials 35 and 70, respectively), and they could ask for additional breaks if they wanted to. The walking part of the study took approximately 60 minutes to complete. After participants had performed their 105 walks, all markers were removed. Participants were debriefed, signed the final consent form and were thanked for participation.

After walking and before debriefing, participants in the “walking whilst performing a memory task” condition were tested to confirm whether they had indeed memorised the images seen. For this, they were again presented with the same image set, but this time randomly intermixed with 100 new images (50 nature, 50 urban) and had to decide as quickly and as accurately as possible which images they had seen earlier during the walking part of the study. Only participants who performed on this task with an overall accuracy of 62% or more were included in data analysis. This part of the study took approximately 15 minutes to complete.

## Data analysis

### 3D motion capture data: Step detection and parametrisation of gait

A pre-processing procedure (QTM’s Automatic Identification of Markers) was applied to the raw gait data recorded with the motion capture system (Qualisys Track Manager, 2018). A low-pass filter (bidirectional 2nd order Butterworth filter with a cut-off frequency of 5Hz) was applied to the raw data of interest, i.e. the foot and hip markers, to remove high frequency noise. During normal walking, we expected steps of a typical length alternating between the left and the right foot. Inconsistency in walking was defined as gait data with missing markers, steps over 1.3m in length or consecutive steps from the same foot. All trials with inconsistent walking were checked for marker labelling errors, and errors were corrected where possible or trials removed if there were missing foot or hip marker data.

Data were truncated for each trial to remove all data from the first 0.5 metres (m) when participants began the trial and the last 2m of captured space when participants slowed down (5m before the wall on which images had been presented). This left the distance of 9.5m for gait analysis per trial in which participants were walking with comparably constant speed. Gait data were extracted, in particular position data of the foot markers, to label individual steps and to calculate walking speed. Steps were defined as the stationary periods for each foot (when the marker moved less than 5cm in 0.1s). The position of a step was determined by the position of the foot marker in the middle of this stationary period. The landing time was therefore defined as the time corresponding to the maximal deceleration of the foot on the Y axis prior to this stationary period, and the lifting time as the point of maximum acceleration on the Y axis post stationary period.

Step length was calculated by subtracting the Y-position of the rear foot from the Y-position of the forward-stepping foot and defined as the distance from the foot marker on one foot to the foot marker on the other foot at landing time. Stride time was defined as the difference between one landing time and the subsequent landing time of the same foot. The first step and last step for each foot were removed from analysis due to an undefined lifting time/start position of the rear foot and an undefined landing time/position of the front foot due to walking distance data truncation. Walking speed (i.e. velocity) was calculated as the distance the hip marker travelled divided by the time taken to complete to walk the 9.5 m distance. Mean step length, stride time and walking speed were calculated for each trial, as well as mean variability in step length and stride time. Note that both step detection and parametrisation of gait were calculated in MATLAB (R2018a).

Behavioural and gait data are available at doi: 10.5523/bris.1eyr9knffaxwj1z7m03pcqzliq.

### Exclusion criteria

Five participants were excluded from analysis due to technical issues with the motion capture system during testing (e.g. missing sensor data, below 80% of data). One further participant was excluded on the basis of their memory performance in the memory compliance check. This left 22 (2 male) participants’ datasets for analysis for the “walking whilst performing a memory task” condition, aged 18–61 years (M = 22, SD = 9.12), and 22 (4 male) participants’ datasets for analysis for the “walking whilst performing the visual discomfort rating task” condition, aged 18–35 (M = 21, SD = 3.85).

## Results

### Behavioural outcomes

#### Memory task

A non-parametric Wilcoxon signed-rank test of performance values for correctly memorised images revealed that participants remembered significantly more urban (M = 81%) than nature images (M = 70%); Z = -3.083, p < 0.05. However, there was no significant difference in median reaction times for the two image types as confirmed by a non-parametric Wilcoxon signed-rank test rank test; Z = -0.503, p > 0.05.

#### Visual discomfort rating task

A Paired Samples t-test revealed that there was no significant difference in subjective visual discomfort ratings between nature scenes (M = 2.95, SD = 0.80) and urban scenes (M = 2.76, SD = 0.88), t(21) = 1.707, p > 0.05).

### Gait kinematics

Repeated measures MANOVAs were conducted on three dependent gait measures, combining the gait data for participants of the two cognitive tasks: mean velocity, mean step length and mean stride time with environment image type as a within subject factor (Nature/Urban/Control) and cognitive task type as a between-subject factor (Memory Task/Visual Discomfort Rating Task). The analysis was conducted in SPSS Statistics 24.

#### Velocity

**Fig 1:**
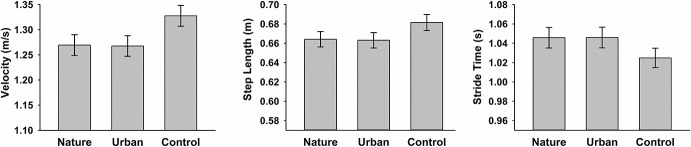
Group (n = 44) averages of individual mean velocity (m/s), individual mean step length (in metres) and individual mean stride time (in seconds) across environment type (nature, urban, control). Error bars reflect ± 1 SEM.

There was a statistically significant main effect of environment on velocity determined by a MANOVA with Greenhouse-Geisser correction, F(1.161, 48.776) = 81.947, p < 0.001, partial η2 = 0.661 (see Fig 1). *Post-hoc* pairwise comparisons using Bonferroni correction revealed that the control condition had a significantly faster walking speed than both nature (p < 0.001) and urban conditions (p < 0.001). Crucially, there was no significant difference between gait velocities obtained for walking during nature and urban conditions (p > 0.05).

#### Step length

There was a statistically significant main effect of environment on step length determined by a MANOVA with Greenhouse-Geisser correction, F(1.173, 49.266) = 70.862, p < 0.001, partial η2 = 0.628 (see Fig 1). *Post-hoc* pairwise comparisons using Bonferroni correction revealed that the control condition had a significantly longer step length than both nature (p < 0.001) and urban conditions (p < 0.001), but nature and urban conditions did not differ from each other (p > 0.05).

#### Stride time

There was a statistically significant effect of environment on stride time determined by a MANOVA with Greenhouse-Geisser correction F(1.336, 47.711) = 65.835, p < 0.001, partial η2 = 0.611 (see Fig 1). As for the two other gait measures, post-hoc pairwise comparisons using Bonferroni correction revealed that the control condition had a significantly shorter mean stride time than both nature (p < 0.001) and urban conditions (p < 0.001) which again did not significantly differ from each other (p > 0.05).

Cognitive task (memory task vs visual discomfort task) did not differentially affect any of the gait measures, nor were there any significant interactions between task and environment.

### Multi-level modelling

Multi-level modelling was applied to cross-classified data from both cognitive tasks (n = 44) to determine the impact of environment, pre-defined image liking scores, image fractal dimensions, and cognitive task on velocity (m/s), in MLwiN 3.03 software. Specifically, we fitted the cross-classified model using the Markov chain Monte Carlo (MCMC) method with the Bayesian Deviance Information Criterion (DIC) to handle more complex cross-classified models [56].

The analysis was run on walking speed (velocity) as dependent variable as it is considered to be the most sensitive of the three gait measures investigated.

Control images were excluded from this analysis due to missing data for pre-defined liking scores and for fractal content. Environment and task variables were dummy coded, and continuous data were transformed into Z-scores.

The multi-level structure treats all trials (N = 4362) as nested within both image and participant. Three variables were classified as random effects, with trial at level 1, and image and participant at level 2 (model 1, see Table 1).

**Table 1:** Model fit comparisons for models with standardised velocity as a dependent variable.

Model	DIC	Fixed	Random
1	4374.070		PT, IM, T
2	4374.418	ENV, LIK, TK, FR	PT, IM, T
2a	4372.084	LIK	PT, IM, T
3	4376.349	LIK, LIK*ENV, LIK*TK, LIK*FR	PT, IM, T
3a	4374.070	Model 1^a^	PT, IM, T

Random effects: PT = Participant, IM = Image, T = Trial. Fixed effects: ENV = Environment, LIK = Pre-defined Liking Score, FR = Fractal Dimension, TK = Task. ^a^ Please note that adding interactions to model 3 revealed that all predictors, including LIK, were insignificant; thus, model 3a equals model 1.

A series of models were fitted through two stages to establish the model of best fit. At the first stage, all predictors (environment, liking score, task and fractal dimension) were added as fixed effects to a cross-classified model (model 2, see Table 1). At the second stage, two-way interactions were added as fixed effects. After each stage, the significance of each fixed effect (predictor) was assessed with chi-squared statistics, and insignificant predictors were discarded. The selection of the best model was based on the Deviance Information Criterion (DIC) statistic. A lower DIC equates a better fit.

Table 1 shows the results for all models fitted; models lettered ‘a’ show the best combination of predictors at each stage, following the removal of insignificant predictors.

The results of the analysis revealed that model 2a was the best fitting model, with predefined liking scores being a significant predictor ($X_{1}^{2}$ = 4.657, p < 0.05) for walking speed: people walked faster towards images with higher liking scores. Parameter estimates for the model are displayed in Table 2.

**Table 2:** Fixed effects estimates (top) and random effect variance estimates (bottom) for the model with best fit (Model 2a; Table 1).

Parameter	Estimate	Std. Error	95% CI Lower	95% CI Upper	$X_{1}^{2}$
***Fixed***					
Intercept	0.023	0.128	-0.285	0.251	0.031
Liking	0.016	0.007	0.001	0.030	4.657*
***Random***					
Participant	0.904	0.205	0.587	1.375	
Image	0.002	0.001	0.001	0.003	
Trial	0.157	0.003	0.150	0.164	
Deviance Information Criterion (DIC)					4372.084

Note. Estimates reflect the size of the effect on standardised velocity. Burn-in (i.e. number of initial iterations discarded) = 500, Chain Length (i.e. number of iterations after burn-in) = 10,000. Degrees of freedom is 1 for all Chi-square ($X_{1}^{2}$) statistics.

*p < 0.05.

#### Visual discomfort

To investigate the potential impact of subjective visual discomfort on walking toward nature and urban images matched for population level pre-determined liking scores, and to be able to compare these data to those of an earlier study [13], we performed a second modelling analysis. For this, we focused on the data of the “walking whilst performing a visual discomfort task” only, applying multilevel modelling to cross-classified data of this task (n = 22) on velocity.

Again, control images were excluded from this analysis due to missing data for pre-defined liking scores, visual discomfort ratings, and fractal content. The “environment” variable was dummy coded and continuous data was transformed into Z-scores.

The multilevel structure treats all trials (N = 2183) as nested within both image and participant. Three variables were classified as random effects, with trial at level 1, image and participant at level 2 (model 1, see Table 3). As before, a series of models were fitted through two stages to establish the model of best fit. At the first stage, all predictors (environment, liking score, subjective visual discomfort and fractal dimension) were added as fixed effects to a cross-classified model (model 2, see Table 3). At the second stage, two-way interactions were added as fixed effects. After each stage, the significance of each fixed effect (predictor) was assessed with chi-squared statistics, and insignificant predictors were discarded.

**Table 3:** Model fit comparisons for models with standardised velocity as a dependent variable.

Model	DIC	Fixed	Random
1	2301.433		PT, IM, T
2	2282.580	ENV, LIK, VD, FR	PT, IM, T
2a	2279.459	VD	PT, IM, T
3	2281.759	VD, VD*ENV, VD*LIK, VD*FR	PT, IM, T
3a	2279.459	Model 2a ^a^	PT, IM, T

Random effects: PT = Participant, IM = Image, T = Trial. Fixed effects: ENV = Environment, LIK = predefined Liking score, VD = subjective Visual Discomfort, FR = Fractal Dimension. ^a^ Please note that adding interactions to model 3 revealed that all added predictors were insignificant (VD*ENV, VD*LIK, VD*FR) while VD was significant; thus, model 3a equals model 2a.

Table 3 shows the results for all models fitted; models lettered ‘a’ show the best combination of predictors at each stage, following the discarding of insignificant predictors.

The results of this analysis revealed that model 2a was the best fitting model: Visual discomfort was a significant predictor $X_{1}^{2}$ = 29.240 p < 0.001, with people walking more slowly towards images they perceived as more uncomfortable to look at. Parameter estimates for the model are displayed in Table 4.

**Table 4:** Fixed effects estimates (top) and random effect variance estimates (bottom) for the model with best fit (Model 2a; Table 3).

Parameter	Estimate	Std. Error	95% CI	95% CI	$X_{1}^{2}$
			Lower	Upper	
***Fixed***					
Intercept	-0.006	0.204	-0.425	0.378	0.001
Discomfort	-0.054	0.011	-0.074	-0.033	29.240***
***Random***					
Participant	0.961	0.329	0.516	1.795	
Image	0.002	0.001	0.000	0.004	
Trial	0.163	0.005	0.154	0.173	
Deviance Information Criterion (DIC)					2279.459

Note. Estimates reflect the size of the effect on standardised velocity. Burn-in = 500, Chain Length = 10,000. Degrees of freedom is 1 for all Chi-square ($X_{1}^{2}$) statistics.

***p < 0.001.

Note that under these conditions pre-defined liking scores were not predictive of velocity changes whilst subjective visual discomfort ratings were. This raises the question how subjective visual discomfort and group-matched liking scores are related. Results of Pearson’s correlation analysis revealed that there was a negative correlation between visual discomfort ratings and liking scores, r^2^ = - 0.497, p < 0.001, with aesthetics scores explaining 6% of the variation in visual discomfort.

## Discussion

Using two different tasks, we showed that, as predicted, environment type did not affect gait kinematics when participants were presented with images of nature and urban scenes matched for liking scores beforehand by an independent sample. It thus seems that cognitive load differences evoked by exposure to urban as opposed to nature images observed in earlier studies [13] do not arise when images are controlled for their population-defined liking scores; i.e. by presenting pairs of images in which the respective nature and urban images had similar aesthetic rating score.

Indeed, in our earlier study [13], we showed that exposure to nature and urban images differ in their cognitive processing requirements, comparable with models such as Ulrich’s Stress Recovery Theory [10] or Kaplan’s Attention Restoration Theory [5, 6]. These differences in cognitive processing requirements could be measured on a trial-by-trial basis, using changes in gait kinematics: gait speed and step length decreased for exposure to urban as compared to nature scenes [13]; however, in this earlier study we controlled images only for image configuration, not liking scores. In the study presented here, not only were there no differences in gait kinematics between the two environment types, but population-defined liking scores explained some of the gait variability found: indeed, pre-defined liking scores were predictive of velocity, with increased liking scores leading to increased velocity regardless of environment image type or task performed. This observation is in line with ideas that the more one likes the environment one is in, the less cognitively demanding it is (see e.g. ART; [6, 18, 57]. That liking scores were predictive of velocity confirms that our analysis itself was sensitive enough to detect systematic changes in gait kinematics; thus, strengthening our conclusion that the lack of processing differences between the two image types is due to having accounted for the impact of aesthetic preference on cognitive processing (e.g. [14]) rather than a lack of sensitivity of our experimental method.

Our findings thus seem to support the idea that liking is a factor underlying environmental benefits, irrespective of whether these are nature or urban environments. It is thus tempting to conclude that any potential differences in cognitive processing between nature and urban environments are rather quantitative than qualitative in nature, in line with other studies in Environmental Psychology that demonstrated that improvement in cognitive functioning occurs if an environment has high aesthetic value irrespective of environment type [5, 6, 8, 12, 18, 19]. Whether our liking-matched nature and urban scenes also match with regard to their restorative factors as proposed in ART [19] will have to be established in future studies.

Whilst at first glance our main null result appears to be robust against alternative interpretations, a closer look at our additional findings allows for alternative interpretations:

First, analysis of the data for the visual discomfort rating task revealed that subjective visual discomfort ratings, rather than pre-defined liking scores or fractal dimensions, were the actual drivers behind the observed velocity changes. In other words, subjective visual discomfort seemed a stronger predictor of gait changes than aesthetic preference or liking, as any impact of pre-defined liking scores was absent when subjective visual discomfort was added as a factor to the multilevel model. Note, however, that we cannot decide on the basis of these findings whether visual discomfort has a direct impact on gait speed, or whether it is simply more difficult to rate images that are more uncomfortable to look at, thus slowing people’s decision making and, as a consequence, their gait. Indeed, even though participants had been asked to rate each individual image only after they had finished their walk, we cannot exclude that participants started to rate the image already during the walk and potentially even reassessed their ratings with distance from the actual image. If visual discomfort instead of the actual decision making process were at the core of gait slowing, results would be in support of Ulrich’s Stress Recovery Theory [9, 10]: viewing images that are more uncomfortable to look at might lead to perceptual distortions and other physiologically unpleasant/aversive symptoms which, in turn, make it more difficult and stressful to approach the evoking stimulus.

Second, it should be noted that visual discomfort and liking scores were not independent of each other but negatively correlated. Before speculating about a possible link between these two variables, it is important to point out that any proper examination of the relationship between liking and discomfort is limited through our design and beyond the scope of this study: visual discomfort ratings for individual images were provided by each individual participant whilst liking scores were average liking scores for each image across an independent observer sample. Further, we measured aesthetic image preference on the basis of liking ratings, not disliking ratings, as formation of liking judgments is spontaneous and associated with affective processing [58] and suggested to be linked to aesthetic preferences for nature (SRT, [9, 10]). Disliking judgments, in contrast, are thought to result from more controlled cognitive processes. Moreover, positive affective judgements (“liking”) and negative affective judgements (“disliking”) are not polar opposites, but appear asymmetrically linked in memory [59]. This raises the question whether disliking and visual discomfort would have been a more appropriate comparison than liking and visual discomfort. Future studies therefore might want to have a closer look at the relationship between aesthetic preferences and comfort and discomfort ratings.

Third, could a look at image statistics shed more light on what affects gait slowing and thus cognitive processing load when looking at different landscapes? Even though there was a significant difference in fractal dimensions between nature scenes and urban scenes, we did not find any evidence that fractal dimensions affected gait kinematics, in contrast to earlier findings by Ho et al. [40]. However, the range of fractal differences between the two environment image types might have been too small to be picked up with our current stimulus set.

Forth, our participant sample consisted primarily of young, healthy female participants. How confident are we then that our results can be generalised to other participant groups? Although gait kinematics differ across the life span and in the presence of a variety of disease conditions, sex differences in preferred walking speed for young, healthy volunteers are so small as to be statistically and physiologically insignificant even in the presence of significant variation in height and body mass [see for a review 60–62]. More importantly, we were interested in *relative* gait changes within participants for different environments, finding that all our participants slowed their gait when exposed to more cognitively demanding environments irrespective of their individually preferred walking speed. Based on calculations by Bakdash & Marusich [53] for the number of repetitions per condition for individual participants and the number of participants in our study, our results should thus be generalisable to over 80% of the population. Crucially, in older participants and those with a variety of disease conditions for whom the impact of cognitive load on gait is known to increase [63], environmental impact should be even more pronounced.

Last but not least, a closer look at our task design is required before drawing final conclusions. In our experiment, participants performed a dual-task, i.e. a motor-cognition interference task (walking whilst memorising images or walking whilst rating images for visual discomfort) whilst being exposed to nature and urban environments. How can we therefore exclude the possibility that rather than environmental differences, it was the interaction between environment type and the demands posed by the cognitive task that masked differences induced by stimulus processing itself? In other words, could it be that memorizing nature images or estimating the amount of visual discomfort of nature images was more cognitively demanding than memorizing urban images / estimating the amount of visual discomfort of urban images; thus balancing out any image type-related cognitive load differences with task-related differences? Such an interpretation of our data seems unlikely: whilst it was indeed more difficult to remember nature images than urban images, visual discomfort ratings are known to be more difficult (i.e. to take longer) for urban images (see e.g. [13, 40]). In other words, any cognitive load induced by the two cognitive tasks should have affected interactions with environment type in opposite directions. However, there was no task effect nor any task environment interaction that would support such an interpretation of our data.

In conclusion, our null hypothesis that cognitive load differences between nature and urban scenes are not present if these two environment types are controlled for liking cannot be rejected. It thus seems to be safe to conclude that controlling for aesthetic preferences and / or visual discomfort is a crucial factor when trying to understand the neural mechanisms underlying cognitive benefits of exposure of one environment over another. Future studies on the cognitive benefits of nature over urban environments should keep in mind that the two environments might not differ qualitatively but only quantitatively, try to avoid aesthetics-related stimulus selection biases, and control for the possible impact of semantic associations on liking and visual discomfort.

## Supporting information

S1 File (DOCX)

## References

[1] Pretty J, Peacock J, Sellens M, Griffin M. The mental and physical health outcomes of green exercise. Int J Environ Health Res. 2005;15(5):319–37. Epub 2006/01/19. doi: 10.1080/09603120500155963 . 16416750

[2] Maller C, Townsend M, Pryor A, Brown P, St Leger L. Healthy nature healthy people: ’contact with nature’ as an upstream health promotion intervention for populations. Health Promot Int. 2006;21(1):45–54. Epub 2005/12/24. doi: 10.1093/heapro/dai032 . 16373379

[3] Sandifer PA, Sutton-Grier AE, Ward BP. Exploring connections among nature, biodiversity, ecosystem services, and human health and well-being: Opportunities to enhance health and biodiversity conservation. Ecosystem Services. 2015;12:1–15. doi: 10.1016/j.ecoser.2014.12.007

[4] Tennessen CH, Cimprich B. Views to nature: Effects on attention. Journal of Environmental Psychology. 1995;15:77–85. doi: 10.1016/0272-4944(95)90016-0

[5] Meditation Kaplan S., Restoration, and the Management of Mental Fatigue. Environment and Behavior. 2001;33(4):480–506. doi: 10.1177/00139160121973106

[6] Kaplan S. The restorative benefits of nature: Toward an integrative framework. Journal of Environmental Psychology. Journal of Environmental Psychology. 1995;15(3):169–82. doi: 10.1016/0272-4944(95)90001-2

[7] Taylor AF, Kuo FE. Children with attention deficits concentrate better after walk in the park. J Atten Disord. 2009;12(5):402–9. Epub 2008/08/30. doi: 10.1177/1087054708323000 . 18725656

[8] Berman MG, Kross E, Krpan KM, Askren MK, Burson A, Deldin PJ, et al. Interacting with nature improves cognition and affect for individuals with depression. J Affect Disord. 2012;140(3):300–5. Epub 2012/04/03. doi: 10.1016/j.jad.2012.03.012 ; PubMed Central PMCID: PMC3393816. 22464936 PMC3393816

[9] Ulrich RS, Simons RF, Losito BD, Fiorito E, Miles MA, Zelson M. Stress recovery during exposure to natural and urban environments. Journal of Environmental Psychology. 1991;11:201–30. doi: 10.1016/S0272-4944(05)80184-7

[10] Ulrich RS. View through a window may influence recovery from surgery. Science. 1984;224(4647):420–1. Epub 1984/04/27. doi: 10.1126/science.6143402 . 6143402

[11] Berto R. Exposure to restorative environments helps restore attentional capacity. Journal of Environmental Psychology. 2005;25(3):249–59. doi: 10.1016/j.jenvp.2005.07.001

[12] Berman MG, Jonides J, Kaplan S. The cognitive benefits of interacting with nature. Psychol Sci. 2008;19(12):1207–12. Epub 2009/01/06. doi: 10.1111/j.1467-9280.2008.02225.x . 19121124

[13] Burtan D, Joyce K, Burn JF, Handy TC, Ho S, Leonards U. The nature effect in motion: visual exposure to environmental scenes impacts cognitive load and human gait kinematics. Royal Society Open Science. 2021;8(1). doi: 10.1098/rsos.201100 33614067 PMC7890511

[14] Bratman GN, Hamilton JP, Daily GC. The impacts of nature experience on human cognitive function and mental health. Ann N Y Acad Sci. 2012;1249:118–36. Epub 2012/02/11. doi: 10.1111/j.1749-6632.2011.06400.x . 22320203

[15] Chatterjee A, Vartanian O. Neuroaesthetics. Trends Cogn Sci. 2014;18(7):370–5. Epub 2014/04/29. doi: 10.1016/j.tics.2014.03.003 . 24768244

[16] Graf LK, Landwehr JR. A dual-process perspective on fluency-based aesthetics: the pleasure-interest model of aesthetic liking. Pers Soc Psychol Rev. 2015;19(4):395–410. Epub 2015/03/07. doi: 10.1177/1088868315574978 . 25742990

[17] Graf LK, Landwehr JR. Aesthetic Pleasure versus Aesthetic Interest: The Two Routes to Aesthetic Liking. Front Psychol. 2017;8:15. Epub 2017/02/15. doi: 10.3389/fpsyg.2017.00015; PubMed Central PMCID: PMC5276863. 28194119 PMC5276863

[18] Kaplan R, Kaplan S. The Experience of Nature: A Psychological Perspective. New York: Cambridge University Press; 1989.

[19] Kaplan S, Berman MG. Directed Attention as a Common Resource for Executive Functioning and Self-Regulation. Perspect Psychol Sci. 2010;5(1):43–57. Epub 2010/01/01. doi: 10.1177/1745691609356784 . 26162062

[20] Ibarra FF, Kardan O, Hunter MR, Kotabe HP, Meyer FAC, Berman MG. Image Feature Types and Their Predictions of Aesthetic Preference and Naturalness. Front Psychol. 2017;8:632. Epub 2017/05/16. doi: 10.3389/fpsyg.2017.00632; PubMed Central PMCID: PMC5408127. 28503158 PMC5408127

[21] Van Hedger SC, Nusbaum HC, Clohisy L, Jaeggi SM, Buschkuehl M, Berman MG. Of cricket chirps and car horns: The effect of nature sounds on cognitive performance. Psychon Bull Rev. 2019;26(2):522–30. Epub 2018/10/28. doi: 10.3758/s13423-018-1539-1 . 30367351

[22] Han K-T. An exploration of relationships among the responses to natural scenes scenic beauty, preference, and restoration. Environ Behav. 2010;42:243–70. doi: 10.1177/0013916509333875

[23] Hartig T, Staats H. The need for psychological restoration as a determinant of environmental preferences. Journal of Environmental Psychology. 2006;26(3):215–26. doi: 10.1016/j.jenvp.2006.07.007

[24] Purcell T, Peron E, Berto R. Why do preferences differ between scene types? Environ Behav. 2001;33:93–106. doi: 10.1177/00139160121972882

[25] Ulrich RS. Aesthetic and Affective Response to Natural Environment. Boston, MA.: Springer; 1983. 85–125 p.

[26] Ulrich RS. Natural Versus Urban Scenes: Some psychophysiological effects. Environment and Behavior. 1981;13(5):523–56. doi: 10.1177/0013916581135001

[27] Valtchanov D, Ellard CG. Cognitive and affective responses to natural scenes: Effects of low level visual properties on preference, cognitive load and eye-movements. Journal of Environmental Psychology. 2015;43:184–95. doi: 10.1016/j.jenvp.2015.07.001

[28] Stamps AE. Mystery, complexity, legibility and coherence: A meta-analysis. Journal of Environmental Psychology. 2004;24(1):1–16. doi: 10.1016/s0272-4944(03)00023-9

[29] Abkar M, Kamal M, Maulan S, Mariapan M, Davoodi SR. Relationship between the preference and perceived restorative potential of urban landscapes. HortTechnology. 2011;21:514–9. doi: 10.21273/HORTTECH.21.5.514

[30] Peron E, Berto R, Purcell T. Restorativeness, preference and the perceived naturalness of places. Medio Ambiente y Comportamiento Humano. 2002;2(1):19–34.

[31] Coburn A, Kardan O, Kotabe H, Steinberg J, Hout MC, Robbins A, et al. Psychological responses to natural patterns in architecture. Journal of Environmental Psychology. 2019;62:133–45. doi: 10.1016/j.jenvp.2019.02.007

[32] Kellert SR, Wilson EO. The Biophilia hypothesis. Washington, D.C: Island Press; 1995. doi: 10.14219/jada.archive.1995.0210

[33] Wilson EO. Biophilia. Cambridge: MA: Harvard University Press; 1984.

[34] Joye Y, Van den Berg A. Is love for green in our genes? A critical analysis of evolutionary assumptions in restorative environments research. Urban Forestry & Urban Greening. 2011;10(4):261–8. doi: 10.1016/j.ufug.2011.07.004

[35] Reber R, Schwarz N, Winkielman P. Processing fluency and aesthetic pleasure: is beauty in the perceiver’s processing experience? Pers Soc Psychol Rev. 2004;8(4):364–82. Epub 2004/12/08. doi: 10.1207/s15327957pspr0804_3 . 15582859

[36] Joye Y, De Block A. ’Nature and I are Two’: A Critical Examination of the Biophilia Hypothesis. Environmental Values. 2011;20(2):189–215. doi: 10.3197/096327111x12997574391724

[37] Joye Y, Steg L, Unal AB, Pals R. When complex is easy on the mind: Internal repetition of visual information in complex objects is a source of perceptual fluency. J Exp Psychol Hum Percept Perform. 2016;42(1):103–14. Epub 2015/09/01. doi: 10.1037/xhp0000105 . 26322692

[38] Spehar B, Clifford CWG, Newell BR, Taylor RP. Universal aesthetic of fractals. Computers & Graphics. 2003;27(5):813–20. doi: 10.1016/s0097-8493(03)00154-7

[39] Bourchtein A, Bourchtein L, Naoumova N. On the Visual Complexity of Built and Natural Landscapes. Fractals. 2014;22(04). doi: 10.1142/s0218348x1450008x

[40] Ho S, Mohtadi A, Daud K, Leonards U, Handy TC. Using smartphone accelerometry to assess the relationship between cognitive load and gait dynamics during outdoor walking. Sci Rep. 2019;9(1):3119. Epub 2019/03/01. doi: 10.1038/s41598-019-39718-w; PubMed Central PMCID: PMC6395667. 30816292 PMC6395667

[41] Hagerhall CM, Purcell T, Taylor R. Fractal dimension of landscape silhouette outlines as a predictor of landscape preference. Journal of Environmental Psychology. 2004;24(2):247–55. doi: 10.1016/j.jenvp.2003.12.004

[42] Penacchio O, Wilkins AJ. Visual discomfort and the spatial distribution of Fourier energy. Vision Res. 2015;108:1–7. Epub 2015/01/13. doi: 10.1016/j.visres.2014.12.013 . 25576380

[43] Attwell D, Laughlin SB. An energy budget for signaling in the grey matter of the brain. J Cereb Blood Flow Metab. 2001;21(10):1133–45. Epub 2001/10/13. doi: 10.1097/00004647-200110000-00001 . 11598490

[44] Hibbard PB, O’Hare L. Uncomfortable images produce non-sparse responses in a model of primary visual cortex. R Soc Open Sci. 2015;2(2):140535. Epub 2015/06/13. doi: 10.1098/rsos.140535; PubMed Central PMCID: PMC4448811. 26064607 PMC4448811

[45] Le ATD, Payne J, Clarke C, Kelly MA, Prudenziati F, Armsby E, et al. Discomfort from urban scenes: Metabolic consequences. Landscape and Urban Planning. 2017;160:61–8. doi: 10.1016/j.landurbplan.2016.12.003

[46] Wilkins AJ, Penacchio O, Leonards U. The Built Environment and Its Patterns: a View From the Vision Sciences. Journal of Sustainable Design and Applied Research in Innovative Engineering of the Built Environment. 2018;6(1):2018.

[47] Mastandrea S, Fagioli S, Biasi V. Art and Psychological Well-Being: Linking the Brain to the Aesthetic Emotion. Front Psychol. 2019;10:739. Epub 2019/04/26. doi: 10.3389/fpsyg.2019.00739; PubMed Central PMCID: PMC6458291. 31019480 PMC6458291

[48] Buijs AE, Elands BHM, Langers F. No wilderness for immigrants: Cultural differences in images of nature and landscape preferences. Landscape and Urban Planning. 2009;91(3):113–23. doi: 10.1016/j.landurbplan.2008.12.003

[49] Kaplan R, Yang B. The perception of landscape style: A cross-cultural comparison. Landscape and Urban Planning. 1990;19:252–61.

[50] Petrova EG, Mironov YV, Aoki Y, Matsushima H, Ebine S, Furuya K, et al. Comparing the visual perception and aesthetic evaluation of natural landscapes in Russia and Japan: cultural and environmental factors. Progress in Earth and Planetary Science. 2015;2(1). doi: 10.1186/s40645-015-0033-x

[51] Abello RP, Bernaldez FG. Landscape preference and personality. Landscape and Urban Planning. 1986;13:19–28. doi: 10.1016/0169-2046(86)90004-6

[52] Amboni M, Barone P, Hausdorff JM. Cognitive contributions to gait and falls: evidence and implications. Mov Disord. 2013;28(11):1520–33. Epub 2013/10/18. doi: 10.1002/mds.25674 ; PubMed Central PMCID: PMC4119872. 24132840 PMC4119872

[53] Bakdash JZ, Marusich LR. Repeated Measures Correlation. Front Psychol. 2017;8:456. Epub 2017/04/26. doi: 10.3389/fpsyg.2017.00456; PubMed Central PMCID: PMC5383908. 28439244 PMC5383908

[54] Zhou B, Lapedriza A, Xiao J, Torralba A, Oliva A. Learning Deep Features for Scene Recognition using Places Database. Advances in Neural Information Processing Systems. 2014;27.

[55] Schroeder MR. Fractals, Chaos, Power Laws: Minutes from an Infinite Paradise. New York: W. H. Freeman; 1991.

[56] Kass RE, Carlin BP, Gelman A, Neal RM. Markov Chain Monte Carlo in Practice: A Roundtable Discussion. The American Statistician. 1998;52(2):93–100. doi: 10.1080/00031305.1998.10480547

[57] Kaplan R. The Nature of the View from Home. Environment and Behavior. 2001;33(4):507–42. doi: 10.1177/00139160121973115

[58] Zajonc RB. Feeling and Thinking: Preferences Need No Inferences. American Psychologist. 1980;35:151–75.

[59] Page CM, Herr PM. An Investigation of the Processes by Which Product Design and Brand Strength Interact to Determine Initial Affect and Quality Judgments. Journal of Consumer Psychology. 2002;12:133–48.

[60] Salbach NM, O’Brien KK, Brooks D, Irvin E, Martino R, Takhar P, et al. Reference values for standardized tests of walking speed and distance: a systematic review. Gait Posture. 2015;41(2):341–60. Epub 2014/12/30. doi: 10.1016/j.gaitpost.2014.10.002 . 25542397

[61] Bruening DA, Frimenko RE, Goodyear CD, Bowden DR, Fullenkamp AM. Sex differences in whole body gait kinematics at preferred speeds. Gait Posture. 2015;41(2):540–5. Epub 2014/12/31. doi: 10.1016/j.gaitpost.2014.12.011 . 25548119

[62] Schimpl M, Moore C, Lederer C, Neuhaus A, Sambrook J, Danesh J, et al. Association between walking speed and age in healthy, free-living individuals using mobile accelerometry—a cross-sectional study. PLoS One. 2011;6(8):e23299. Epub 2011/08/20. doi: 10.1371/journal.pone.0023299; PubMed Central PMCID: PMC3154324. 21853107 PMC3154324

[63] Woollacott M, Shumway-Cook A. Attention and the control of posture and gait: a review of an emerging area of research. Gait Posture. 2002;16(1):1–14. Epub 2002/07/20. doi: 10.1016/s0966-6362(01)00156-4 . 12127181

