# Supplementary material for: Nature benefits revisited: Differences in gait kinematics between nature and urban images disappear when image types are controlled for likeability
Source: PLoS One. 2021 Aug 27;16(8):e0256635. doi: 10.1371/journal.pone.0256635 (PMC8396763; doi:10.1371/journal.pone.0256635)
Supplement: S1 File — (DOCX) [file pone.0256635.s001.docx]

**S1 File.**

**Materials and Methods**

**Stimuli**

In two online studies, participants were asked to rate images of photographic environmental scenes for their likeability on 7-point Likert scales. Images consisted of 200 nature and 200 urban scenes, which were equally distributed across the two studies. Scenes had been selected from the “places” category of the scene recognition database [1], in addition to containing photographs of landscape and urban spaces taken in Europe and Australia by the authors. Images presented environmental scenes where people and animals were not visible and varied substantially across landscape types, lighting conditions, colours or viewing angles. Image resolution was 1280x800 pixels.

**Participants**

Participant demographics for the two studies are summarized in table 1:

|  | Study 1 | Study 2 |
| --- | --- | --- |
| Age range (and mean age) | 18-81 (31 years) | 17-66 (31 years) |
| Gender | 60 males, 90 females | 76 males, 72 females, 2 gender not disclosed |
| Migraine | 33 | 17 |
| Grew up in a city (> 100.000 inhabitants) | 67 | 70 |
| Grew up in the town (< 100.000 inhabitants) | 58 | 55 |
| Grew up in the countryside | 28 | 22 |
| Grew up in two places | 2: city and countryside,  1: city and town | 1: city and town,  1: countryside and town |
| Places where participants grew up | Africa (1), America (18), Asia (2), Australia (1), Europe (123), America-Asia (1), America-Europe (3), Prefer not to say (1) | Africa (1), America (25), Asia (9), Europe (108), Africa-America (1), Asia-Europe (3), Australia-Europe (1) Prefer not to say (1) |

All participants reported normal or corrected-to-normal visual acuity. All participants were asked to read an information sheet and to provide consent prior to the beginning of the online study. Participants either volunteered by responding to social media announcements or were recruited via Prolific and reimbursed for their time. The experiment was approved by the Faculty of Life Sciences’ Ethics Committee at the University of Bristol (ref. 2410201876401).

**Material and Task**

The two online studies were both run on the ‘Gorilla’ Platform with the same procedure but different image sets. Each image set consisted of 200 images of which 100 were nature images and 100 were urban images.

At the beginning of the study, participants were asked to fill in a form requesting their demographics (see Table 1).

For the actual task, participants looked at the images, one at a time, presented in random order, and rated each image for its likability on a 7-point Likert Scale: “How much do you like the image?” from ‘1 – Not at all’ to ‘7 – Very much’.

There was one break during each study halfway through, i.e. after 100 trials.

The results of independent t-tests are summarized in table 2:

|  |  | **Nature** (Mean±SD) | **Urban** (Mean±SD) | **Comparison** |
| --- | --- | --- | --- | --- |
| Liking (7-point Likert Scale) | Study 1 | 4.86 ± 0.80 | 3.59 ± 0.84 | t(198)=10.88, p < .001 |
|  | Study 2 | 4.75 ± 0.79 | 3.44 ± 0.92 | t(193)=10.76, p < .001 |
|  | **Total** | **4.81** ± **0.80** | **3.50 ± 0.88** |  |
|  |  |  |  |  |

Images of nature scenes had significantly higher liking scores than images of urban scenes in both studies.

**Matching images of nature and urban scenes for liking scores**

To create the image set for our main study, we used the outcomes of the two studies to select 50 image pairs of nature and urban scenes that had received similar mean liking scores (and, where possible, similarly small liking variance) within the same sample; if two images of the same environment type had similar liking scores and variance, other selection criteria such as viewing angle were considered in addition. This resulted in a stimulus set of 50 image pairs with liking scores ranging between 2.82 and 5.61. An independent samples t-test confirmed that for the final stimulus set there was indeed no significant difference in mean liking scores between nature and urban images (Nature: M = 4.22, SD = .67, Urban: M = 4.22, SD = .67, t(98)= .001 p > .05, mean difference= 0.00013).

**References**

1. Zhou B, Lapedriza A, Xiao J, Torralba A, Oliva A. Learning Deep Features for Scene Recognition using Places Database. Advances in Neural Information Processing Systems. 2014;27.
